# Assessment of knowledge of mental illness in a non-clinical population of tunisian students

**DOI:** 10.1192/j.eurpsy.2021.1053

**Published:** 2021-08-13

**Authors:** B. Abassi

**Affiliations:** Psychiatry E, Razi hospital, Manouba, Tunisia

**Keywords:** Mental Health Knowledge Schedule scale, knowledge, Mental illness, Stigma

## Abstract

**Introduction:**

Stigma, including beliefs about mental illness, can operate in different cultures in different ways, making Western theoretical bases considered “universal” on the stigmatization of theories not applicable to non-Western cultures; hence the need for international studies on this subject.

**Objectives:**

This work aimed to assess knowledge of mental illness, available treatments and recovery in a non-clinical sample of Tunisian university students.

**Methods:**

In a cross-sectional descriptive study from October 1 to November 30, 2019, we evaluated 714 students from 3 Tunisian public universities using the Mental Health Knowledge Schedule scale (MAKS).

**Results:**

We found that 34.2% of students did not agree that drugs can be an effective treatment for people with mental health issues, while 76.4% agreed on the effectiveness of psychotherapy. In addition, 34.3% did not consider drug addiction as a mental illness and 21.9% did not consider depression as a mental illness. We objectified a significant correlation of the MAKS score with gender (p=0.019), living environment (p=0.001), high academic level of father (p=0.000) and mother (p=0.027) and presence of personal psychiatric history (p=0.013).

**Conclusions:**

Awareness and information campaigns aimed at developing the general public’s knowledge of the scientific, medical and psychosocial causes of mental illness and the means of management should be established.

